# Editorial: Mechanisms of plant host resistance against viruses

**DOI:** 10.3389/fpls.2024.1506089

**Published:** 2024-10-22

**Authors:** Régis L. Corrêa, Marko Petek, Maite F. S. Vaslin

**Affiliations:** ^1^ Institute for Integrative Systems Biology (I2SysBio), Consejo Superior de Investigaciones Cientificas (CSIC) - Universitat de València (UV), Paterna, Spain; ^2^ Department of Genetics, Federal University of Rio de Janeiro (UFRJ), Rio de Janeiro, Brazil; ^3^ Department of Biotechnology and Systems Biology, National Institute of Biology, Ljubljana, Slovenia; ^4^ Department of Virology, Federal University of Rio de Janeiro (UFRJ), Rio de Janeiro, Brazil

**Keywords:** plant virus resistance, transcriptome (RNA-seq), plant hormonal signaling, RNA silencing, disease resistance, virus-induced gene silencing (VIGS), CRISPR/Cas9, host-pathogen interaction

Plants are the foundation of world health and economic prosperity. They feed billions of people, with main crops such as rice, wheat, and corn serving as the basis of global meals. Ecological disturbances, together with land-use policies and global trades, are promoting the emergence or reemergence of different pathogens. Due to their high evolvability, viruses are among the most frequent emerging pathogens in plants, causing moderate to severe effects on plant production, costs, and yields (Parrella et al., 2022). Despite decades of improvement in their control, viral infections remain extremely difficult to eradicate. Measures are typically limited to phytosanitary treatments or the use of chemical agents to eliminate vectors, which significantly raises expenses and, in some cases, harms the environment and farmers’ health. To address this challenge, innovative, precise, and fast biotechnological tools are being advocated for use in agriculture alongside existing ones to create climate-resilient plants (Ray et al., 2012). Understanding how plants and viruses interact is therefore critical for developing new strategies to generate virus-resistant or -tolerant varieties.

The Research Topic “Mechanisms of Plant Host Resistance Against Viruses” seeks to bring together current and relevant contributions to the field of plant virus interactions and resistance mechanisms. A collection of publications on novel virus diseases (Favara et al.; Wang et al.), contrasting host responses to infection (Xu et al.; Kumar et al.), resistance mechanism manipulation (Liu et al.; Lu et al.; Hayashi et al.), and virus-directed targeting approaches (Delgado-Martín et al.) are discussed.

Virus identification and symptom analysis are crucial for improving our understanding of plant-virus interactions and developing effective, long-term resistance methods to protect plants from viral infection. Favara et al. investigated the interaction between *Plumeria pudica* and the orthotospovirus groundnut ringspot virus (GRSV). The study described the first virus associated with this ornamental plant. Infected plants showed intermittent symptom manifestation, consistent with their known natural resilience to viruses. Interestingly, symptomatic branches, but not asymptomatic ones, frequently resulted in plants showing GRSV symptoms when propagated vegetatively. The implicated restriction in viral systemic movement may further aid our understanding of plant-virus relationships. Wang et al. also tracked host responses to a newly identified disease. A systems biology approach was used to demonstrate that poplar plants infected with the potyvirus bean common mosaic virus (BCMV) exhibit significantly altered transcriptional and posttranscriptional regulation, resulting in general decrease in physiological performance. The findings improve our understanding of how poplar trees respond to viral infections and suggest potential techniques for increasing resistance in poplar and other plant species.

Using plants with contrasting resistance to pathogens provides a powerful approach for dissecting the molecular mechanisms of plant immunity, as it allows for the identification of critical genes, pathways, and temporal patterns that underlie effective defense strategies. A time course transcriptome study was performed by Kumar et al. on watermelon cultivars with varying susceptibility to the whitefly-transmitted squash vein yellowing virus (SqVYV). Their results showed that throughout the infection period, the resistant cultivar had higher levels of the plasmodesma callose binding protein gene, along with several RNA silencing and phytohormone-related genes, than the susceptible cultivar. Conversely, genes related to cell wall and growth were activated in the susceptible cultivar during virus infection. Transcriptomic approaches were also used by Xu et al. to uncover resistance pathways in virus-infected tobacco cultivars having contrasting susceptibility to nematodes. Interestingly, tobacco plants resistant to root-knot nematode disease (RKN) exhibit an hypersensitive response when infected with potyvirus Y (PVY). RKN-resistant and -susceptible plants had over 8,000 differently expressed transcripts 7 days after PVY infection. RKN-susceptible plants can also prevent virus transmission and replication by activating critical pathways such as sucrose and starch metabolism, energy synthesis, and auxin signaling. RKN-resistant plants on the other hand appear to combat virus infection by upregulating genes involved in phenylpropanoid biosynthesis, abscisic acid (ABA), salicylic acid, brassinosteroids, and the jasmonic acid (JA) signaling pathway. This knowledge can be applied to enhance plant resistance in agriculture and expand our understanding of host-pathogen interactions.

Building upon foundational mechanistic understanding of plant-virus interactions, research of strategic manipulation of pathways to enhance resistance against plant viruses is presented in this Research Topic. Hayashi et al. used segregation analysis of crossed TYLCV resistant and susceptible *Nicotiana benthamiana* strains followed by CRISPR-based disruption of RNA-dependent RNA polymerase genes and found that these genes are not responsible for TYLCV resistance. Their work suggests that an unidentified TYLCV resistance gene is present in the wild accession of *N. benthamiana*, which if identified, would be important for breeding tomato resistance against TYLCV. Using CRISPR/Cas9 to disrupt *OsV-ATPase d*, a gene encoding a subunit of vacuolar proton pump, Lu et al. produced transgenic rice with enhanced resistance against Southern rice black-streaked dwarf virus (SRBSDV). Transcriptomics analysis revealed that the enhanced resistance is mediated through JA and ABA immune response pathways. Liu et al. have shown that upregulating the ethylene biosynthesis gene *ClACO5* or its direct regulator *ClWRKY70* enhances plant responses to cucumber green mottle mosaic virus (CGMMV) in watermelon (*Citrullus lanatus*). They confirmed the roles of these genes by overexpression and virus-induced gene silencing experiments in *N. benthamiana.* In a different approach, Delgado-Martín et al. investigated the efficacy of double-stranded RNA (dsRNA) directly targeting virus genes as a defense generation strategy. They discovered that delivering dsRNA against CGMMV by spraying or agroinfiltration effectively protected cucumbers from this severe threat to cucurbit crops. These examples solidify the potential of biotechnological approaches such as gene silencing and CRISPR/Cas9 to engineer future-proof virus-resistant crops.

The research in this Research Topic is paving the way for the integration of mechanistic understanding of plant-virus interactions into the engineering of resilient crop varieties that can effectively counter viral threats and adapt to evolving environmental challenges. Such crops will play a pivotal role in ensuring global food security and sustainable agriculture.
